# Dysfunctional connectivity as a neurophysiologic mechanism of disorders of consciousness: a systematic review

**DOI:** 10.3389/fnins.2023.1166187

**Published:** 2023-07-19

**Authors:** Gabriela Plosnić, Marina Raguž, Vedran Deletis, Darko Chudy

**Affiliations:** ^1^Department of Pediatrics, University Hospital Centre Zagreb, Zagreb, Croatia; ^2^Department of Neurosurgery, Dubrava University Hospital, Zagreb, Croatia; ^3^School of Medicine, Catholic University of Croatia, Zagreb, Croatia; ^4^Albert Einstein College of Medicine, New York, NY, United States; ^5^School of Medicine, University of Zagreb, Zagreb, Croatia

**Keywords:** disorders of consciousness, functional connectivity, disconnection syndrome, fMRI, EEG, neuromodulation

## Abstract

**Introduction:**

Disorders of consciousness (DOC) has been an object of numbers of research regarding the diagnosis, treatment and prognosis in last few decades. We believe that the DOC could be considered as a disconnection syndrome, although the exact mechanisms are not entirely understood. Moreover, different conceptual frameworks highly influence results interpretation. The aim of this systematic review is to assess the current knowledge regarding neurophysiological mechanisms of DOC and to establish possible influence on future clinical implications and usage.

**Methods:**

We have conducted a systematic review according to PRISMA guidelines through PubMed and Cochrane databases, with studies being selected for inclusion via a set inclusion and exclusion criteria.

**Results:**

Eighty-nine studies were included in this systematic review according to the selected criteria. This includes case studies, randomized controlled trials, controlled clinical trials, and observational studies with no control arms. The total number of DOC patients encompassed in the studies cited in this review is 1,533.

**Conclusion:**

Connectomics and network neuroscience offer quantitative frameworks for analysing dynamic brain connectivity. Functional MRI studies show evidence of abnormal connectivity patterns and whole-brain topological reorganization, primarily affecting sensory-related resting state networks (RSNs), confirmed by EEG studies. As previously described, DOC patients are identified by diminished global information processing, i.e., network integration and increased local information processing, i.e., network segregation. Further studies using effective connectivity measurement tools instead of functional connectivity as well as the standardization of the study process are needed.

## Introduction

Consciousness is frequently described as a compound phenomenon and still represents one of the major scientific challenges. In the beginning, many efforts have been made to find the neuroanatomical correlate of consciousness. Unfortunately, it is not that simple. Consciousness is a process rather than an object, and its main characteristic is “*one’s sense of being a unified person despite being confronted with a diversity of sense impressions from different sense organs*” (Smythies et al., 2013). Among number of theories that have been suggested to elaborate the phenomenon of consciousness is neuronal oscillation theory (Seth and Bayne, 2022). It may be the one that provides the best description of the process itself. Vice versa, disrupting these processes, i.e., functional disconnection, plays a critical role the pathogenesis of disorders of consciousness (DOC) (Fernández-Espejo et al., 2012). In this systematic review, we gather evidence regarding patterns of dysfunctional connectivity in patients with disorders of consciousness.

Basically, consciousness represents the state of self-awareness and environment awareness (Laureys et al., 2010). Conscious behavior requires both wakefulness and content awareness, such as cognitive, affective, or sensory experience. Traumatic brain injury is a disastrous incident causing disruption of the brain’s arousal and awareness systems, moderated by the brainstem and cortex. The severest injuries result in prolonged DOC consisting of the vegetative state (VS) and the minimally conscious state (MCS). In addition, VS is recently referred as post-coma unawareness or unresponsive wakefulness syndrome (UWS) (Laureys et al., 2010). Diagnosis of VS is based on “*no evidence of self or environment awareness, as well as absence of sustained, reproducible, purposeful, or voluntary behavioural response to visual, auditory, tactile, or noxious stimuli and language comprehension or expression*” (Multi-Society Task Force on PVS, 1994a,b). The MCS is presented by “*partial preservation of awareness of self and environment, responding intermittently but reproducibly to verbal command and demonstrating some degree of basic language comprehension*” (Giacino et al., 2002). Moreover, the MCS has been additionally classified in the MCS minus and MCS plus state, indicating MCS plus patients as one who can intelligibly or intentionally verbalize and communicate (Bruno et al., 2011a). The confusion in the diagnosis of MCS and VS could be done in patients with locked-in syndrome (Bruno et al., 2011b). The patients in locked-in syndrome are awake and conscious but selectively deafferented by lesion of corticospinal and corticobulbar pathways. They cannot speak, move limbs, or have facial movements (Bodart et al., 2013).

The possibility of consciousness recovery depends on the brain destruction degree and lesion etiology; still, after a year of unresponsive behavior, odds for recovery decrease (Multi-Society Task Force on PVS, 1994a,b; Giacino et al., 2014).

Over decades, a number of different clinical scales have been used to classify DOC patients. The latest scale to evaluate consciousness state nowadays is the JFK Coma Recovery Scale-Revised (CRS-R), based on the Disability Rating Scale (DRS) and the Coma Recovery Scale (CRS), including scoring of auditory, visual, motor, verbal functions, responsiveness, and arousal (Kalmar and Giacino, 2005). CRS-R total sum ranges from 0 (worst) to 23 (best), with specific subscores revealing MCS minus, MCS plus, or emergence MCS form. Similar to the DRS, the Coma/Near Coma (CNC) scale is associated with patients health status, course of treatment, outline and to the fundamental neurophysiological impairment (Rappaport, 2005).

Although effective treatment for this group of patients is not yet available, some progress has been made by introducing neuromodulation techniques, in the first-place deep brain stimulation (DBS). Historically, Morruzi and Magoun first showed brainstem reticular formation and thalamus stimulation of the anesthetized animals leading to desynchronization of low-frequency disorganized electroencephalograph (EEG) activity and background activity comparable to the patterns in wakeful states (Moruzzi and Magoun, 1949). Mentioned experiments, alongside other findings, aimed promote the concept of a reticular ascending activating system (RAAS) controlling sleep–wake cycle (Tapia et al., 2013). DBS emerged in 1960 as a potential therapeutical method and since then has been used in the thalamus, upper brainstem, high spinal cord, and associated targets in the basal ganglia (nowadays mainly in the central thalamic nucleus) in attempts to restore consciousness (Chudy et al., 2020).

In mentioned primary, as well as in later studies, the vast majority of patients manifest eye-opening and incomplete movements when receiving stimulation consistent with an arousal effect. Nevertheless, arousal effect occurrence did not predict any improvement. Furthermore, arousal effect, including eye-opening, autonomic function changes and EEG desynchronization characterize a fundamental and wide activation of the forebrain, brainstem, and spinal cord systems. It seems that apparent wakefulness and incomplete movements do not demonstrate higher integrative brain function recovery – it just gives evidence that DBS electrodes hit the target (Chudy et al., 2018).

Several research groups are continuously making efforts to improve patient selection criteria for DBS as well as neurostimulation protocols. The inclusion criteria for such procedures traditionally require the presence of multimodal evoked potentials (somatosensory, motor and visual evoked potentials), which are believed to be gross neurophysiological markers of cortical functional integrity.

Today, however, with the rapid advances in computing and neuroimaging techniques to test brain connectivity, we are faced with enormous amounts of data and novel mathematical modeling techniques. All this information might improve our understanding of underlying pathophysiological processes and lead us to better decision-making, but the careful interpretation is crucial.

Given that the estimated number of glia and neuronal cells in the human brain is around 10^11^ and there are roughly 10^14^ synaptic connections, it is impossible (and even pointless) to investigate disrupted connectivity patterns at a single neuron level (Dicke and Roth, 2016; von Bartheld et al., 2016). Therefore, the common goal is to make as precise as possible approximations of neuronal interactions while trying to avoid data overwhelming. Before even doing so, it is necessary to take a well-organized approach to different scale neuronal units within the appropriate space and time frame. Moreover, the fMRI’s high spatial resolution and the EEG’s high temporal resolution are complementary for understanding neural processes (Itthipuripat et al., 2019).

Therefore, the aim of this paper is a systematic review of conceptual framework derived from fMRI and EEG studies on DOC patients, current advances in the understanding of DOC pathophysiology and diagnostic role of neuromodulation protocols.

## Methods

### Search strategy

We have conducted a systematic review according to PRISMA guidelines (Page et al., 2021). The search was done on articles published up to January of 2023. We searched the PubMed and Cochrane databases for articles using the following keywords: “disorders of consciousness” and “functional connectivity,” “vegetative state” and “functional connectivity,” “unresponsive wakefulness syndrome” and “functional connectivity” and “minimally conscious state” and “functional connectivity.”

**Figure 1:**
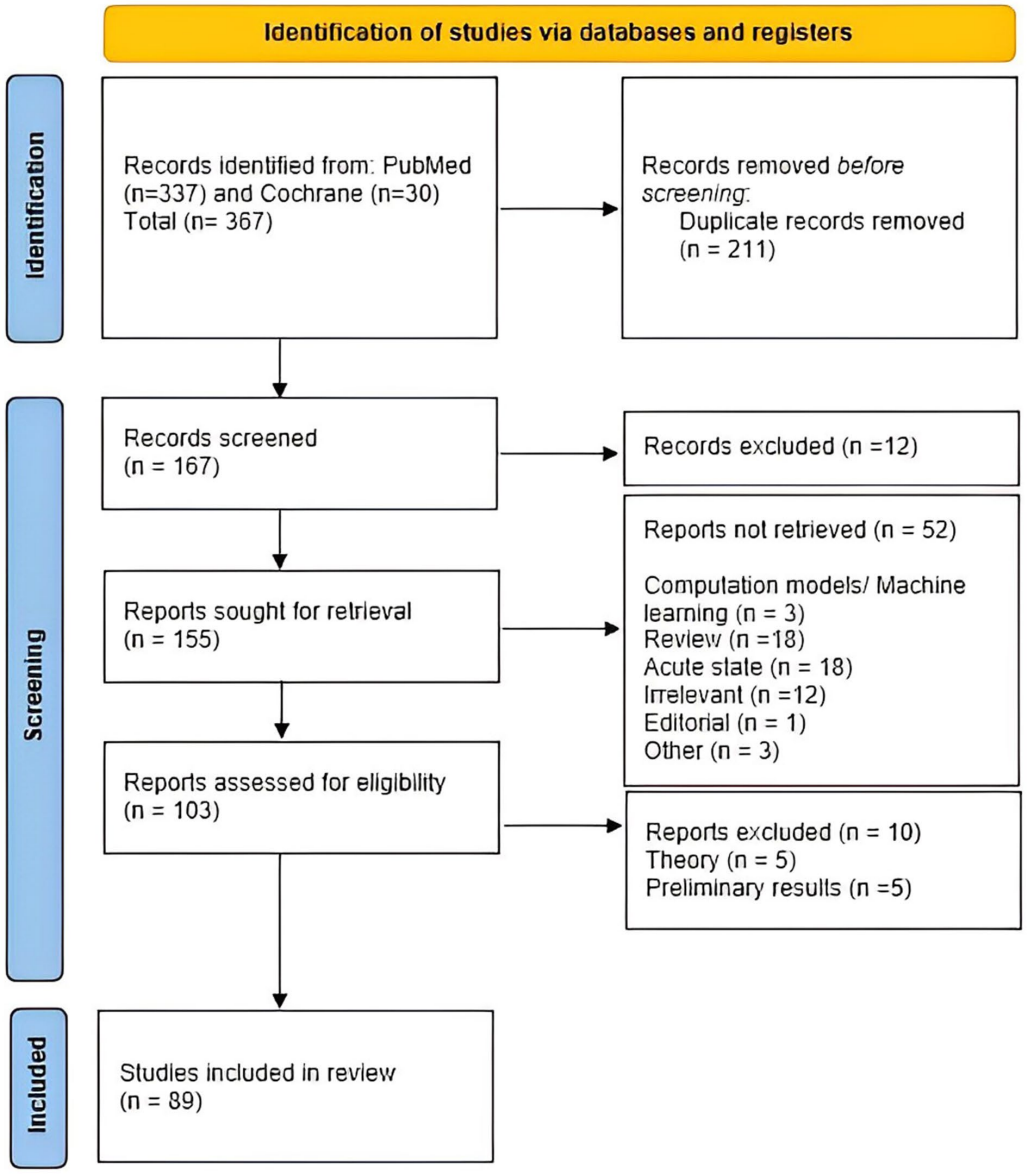
Prisma flow chart of the systematic review.

After we applied appropriate filters, the search rendered 367 records. The studies were then selected based on the following inclusion and exclusion criteria (Figure 1). Articles were first screened by title and abstract, followed by full-text checking for their eligibility. Four authors selected the articles independently (PG, RM, CD, and DV), and final inclusion was done by agreement.

### Inclusion and exclusion criteria

Studies accepted for inclusion were: (a) studies with patients diagnosed with DOC; (b) studies published up to January of 2023; (c) published in the English language; (d) published in indexed and peer-reviewed journals; and (e) evaluated consciousness using validated scales and scoring systems (usually by CRS-R scale).

Exclusion criteria include: (a) studies published in regional languages other than English, (b) examination in an acute state or during sedation/anaesthesia, and (c) no clear methodology or testing parameters described. Additionally, we excluded papers on basic research, brain-computer interfaces, machine learning, or pharmacological treatment response.

Studies were checked for quality, and finally, 89 studies were included (Figure 1).

### Conceptual frameworks for DOC research

**Figure 2:**
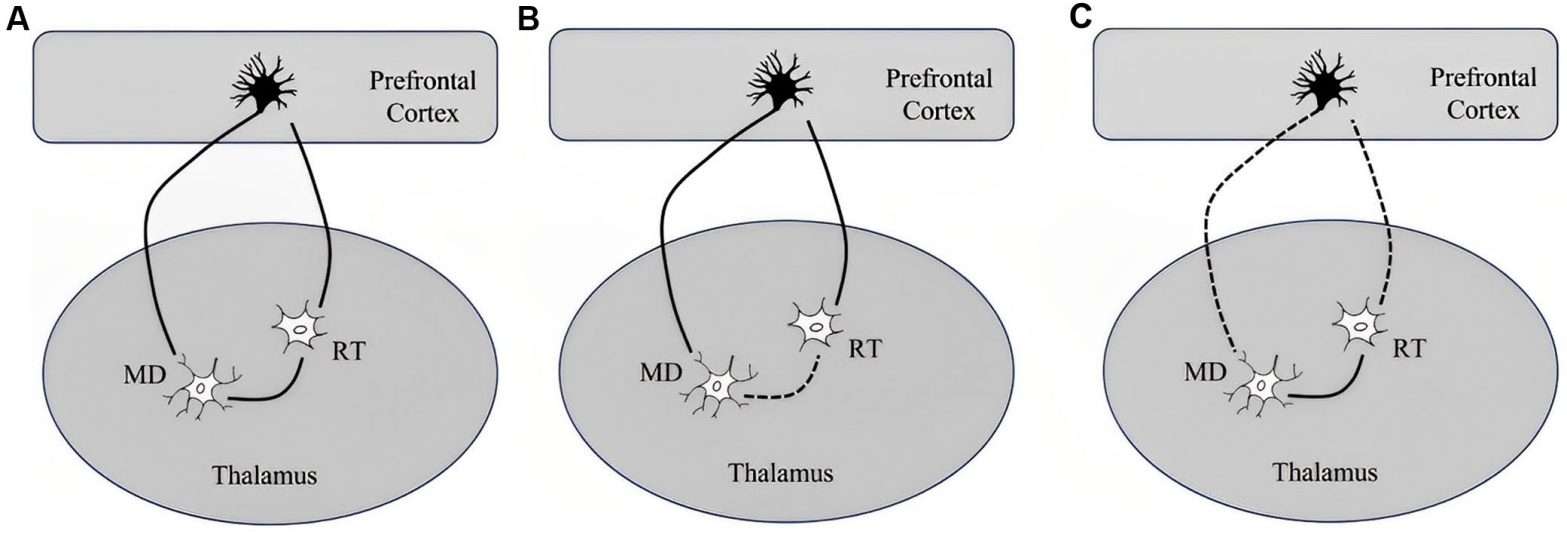
Simplified illustration of network including a thalamic relay neuron (MD), reticular neurons (RT), and a corticothalamic pyramidal neuron and their response to a short or long duration of cardiac arrest according to Ross and Graham study (Ross and Graham, 1993): **(A)** network segregation with intact RT and MD, **(B)** network integration with selective degeneration of RT following a short-term ischemia leaving cortico-thalamic reticular and thalamo-cortico-reticular innervation preserved, and **(C)** dysfunctional network organization in DOC characterized by impaired network integration, increased network segregation and topological reorganization with selective sparing of the RT following long-term complete ischemia causes the death of corticothalamic pyramidal neurons and thalamic relay neurons in the MD.

We find increasing evidence linking DOC with interference in brain connectivity both locally and connecting remote brain areas. Disconnection generally indicates brain dysfunction following lesions to white matter connections (Catani and Ffytche, 2005). Theoretically speaking, consciousness has two different and separated components, both level and content (Laureys, 2005). While the consciousness level describes the extent of arousal or wakefulness, the content portray subjective experience or awareness. Anatomically, it is feasible to connect wakefulness with thalamocortical, vertical connectivity, while awareness rely on cortico-cortical, horizontal connectivity (Modolo et al., 2020). Wakefulness depends critically on thalamocortical connectivity, which is highly dependent on RAAS. Neurons of the human thalamic reticular nucleus (RT) are considered selectively vulnerable to ischemic neuronal damage following cardiac arrest (Ross and Graham, 1993) (Figure 2).

So, despite severe and irreversible damage of RT, cortical neurons and their connections with the thalamus may still be preserved. These pathological findings explain the fact that in some patients with severe DOC (VS and MCS), cortical somatosensory evoked potentials can still be elicited. RAAS bind together different cortical regions, and ischemic injury selectively lesson RAAS while leaving some cortical neurons intact, resulting in loss of consciousness of various degrees. On the other hand, awareness is mostly related to frontoparietal associative cortices. It is later subdivided into an internal awareness network (mostly midline regions), and an external awareness network (mostly lateral frontoparietal hemispheric regions).

**Figure 3:**
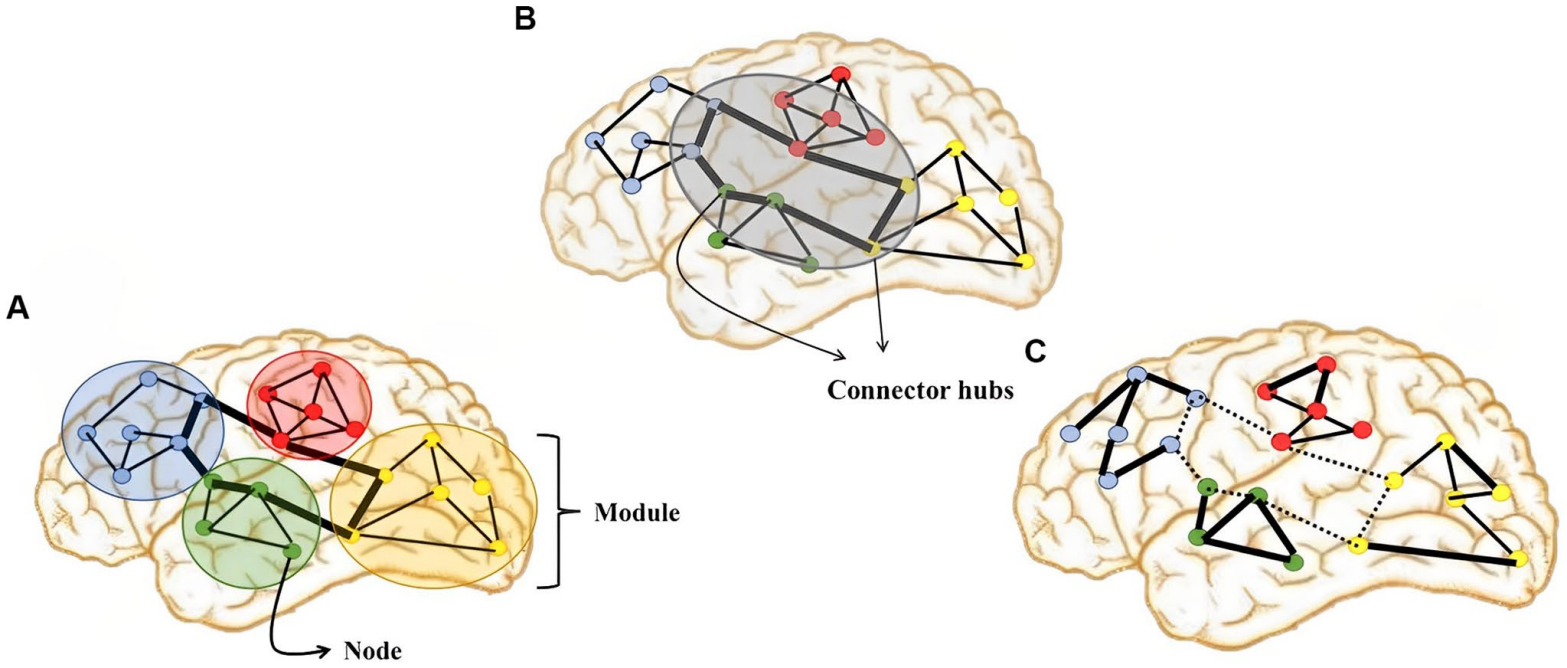
Illustration of graph-theoretic measures showing: **(A)** network segregation, **(B)** network integration, and **(C)** dysfunctional network organization in DOC which is characterized by impaired network integration, increased network segregation and topological reorganization.

In the last decades, an extraordinary flourishing of structural and functional imaging techniques, combined with mathematical models, offered us new ways for further advances in lesion mapping. Network theory is an umbrella term due to mathematical theory for the networks description. Therefore, a number of non-invasive novel techniques and methods analysis have provided whole-brain connectivity patterns inspection using electrical and magnetic brain activity (i.e., EEG, MEG), as well as cerebral blood flow changes as quantification of neural activity (fMRI) for network construction (Stephan et al., 2000). Thus, the network nodes are EEG electrodes, MEG sensors, and fMRI voxels or regions of interest, containing complex signal of the neurons activity (Figure 3).

To describe the relationship between pairs of network nodes, the term functional connectivity (FC) has been introduced. However, it is essential to point out that functional connectivity does not necessarily correspond to structural (anatomical) connections. Understanding the relationship between structural and functional organization represents one of the most critical challenges in neuroscience. In contrast to functional connectivity, effective connectivity includes information about the direction of the connection. Even so, present-day fMRI resting-state methods for causal connectivity are limited (Ramsey et al., 2014).

The network model is convenient for describing static brain properties, but another mathematical model is introduced, the so-called neuronal oscillations model, to evaluate dynamic interactions. Neural oscillations are widespread phenomena ranging from the microscopic level of individual neuron electric state oscillations to large neuronal ensembles macroscopic oscillations.

The recuring presynaptic neurons firing generates oscillatory activation, while the synchronized activity of several neurons generate macroscopic oscillations. Mentioned was first observed by Hans Berger in 1924 in EEG, leading to the brain rhythms classification into frequency bands. Rather than using single-neuron models, it is helpful to emerge a low-dimensional models to imitate the assembly of number of near-identical interconnected neurons with a preference to operate in synchrony. These neural mass models are composed out of state variables that track coarse-grained measures of the average membrane potential, firing rates, or synaptic activity (Ashwin et al., 2016).

"*What fires together, wires together*" is a well-known (but simplified) Hebb's principle and, while mnemonic, could be very misleading: if neurons activations occur at the same time, the activation of one neuron cannot cause activation of the other. The actual ' 'Hebb's quote was: *“when an axon of cell A is near enough to excite a cell B and repeatedly or persistently takes part in firing it, some growth process or metabolic change takes place in one or both cells such that A's efficiency, as one of the cells firing B, is 'increased”*. An attentive reveals the principle of causality and consistency (Keysers and Gazzola, 2014).

## Results

### Structural neuroimaging changes

Structural imaging approaches have not identified specific and consistent focal abnormalities, but there is evidence of diffuse irregularity in volume of both grey and white matter and connectivity in patients with disorders of consciousness (DOC) (Snider and Edlow, 2020). Diffusion MRI, usually processed with tractography, is widely used in DOC patients to investigate the integrity of white matter, i.e., structural connectivity (Snider and Edlow, 2020). Recently, ultra-high-field MRI at 7 Tesla in combination with Graph-theoretical analysis and network-based statistics was introduced to explore the structural network and white matter microstructure alterations (Tan et al., 2019). Network-based statistical analysis revealed significantly decreased structural connectivity, mainly in the frontal cortex, limbic system, occipital, and parietal lobes (Tan et al., 2019).

### Functional neuroimaging changes

The signals obtained from PET, fMRI, and fNIRS rely on the detection of localized alterations in cerebral blood flow that are linked to neural activity. Functional neuroimaging investigations in DOC can be categorized into two types: resting-state studies and stimulus-based studies (Supplementary material). Resting-state functional connectivity, which does not require active participation, is especially convenient in this group of patients (Soddu et al., 2015).

The introduction of fMRI has brought significant advancements, with the majority of studies on patients with DOC relying on fMRI. While fMRI has limited temporal resolution (within a few seconds), it offers excellent spatial resolution (within millimeters). PET, despite being a more robust technique than fMRI, seems to be more sensitive than active fMRI in aiding the clinical diagnosis of DOC patients, although it may have led to a higher rate of false positives (Stender et al., 2014; Soddu et al., 2015).

Historically, the analysis of brain networks has focused on their anatomical organization. However, there is a recent emerging trend that highlights the importance of examining the topological aspects of these networks. Topological metrics allow us to understand the relationships between elements within a system, irrespective of their physical placement. In this section, we will first discuss the patterns of dysfunctional connectivity observed in the anatomical organization of brain networks, followed by an exploration of the subsequent topological reorganization. PET has been used to assess cerebral metabolic activity in DOC patients and has shown modified connectivity between intralaminar nuclei of the thalamus and prefrontal and anterior cingulate cortices in the VS, while not after consciousness recovery (Laureys et al., 2000). Hypermetabolism in the RAAS and impaired functional connectivity between the RAAS and the precuneus have also been reported (Boly et al., 2009).

Lacking cortico-thalamic and reduced cortico-cortical connectivity patterns were also described in fMRI studies (Boly et al., 2009), as well as a considerable decrease in both specific and nonspecific thalamic functional connections (Zhou et al., 2011).

Depending on the analysis method, there are several strongly connected resting state neural networks (RSNs) of sensory and cognitive relevance including the default mode network (DMN), somatomotor network, dorsal attention network, ventral attention network, limbic system network, fronto-parietal and visual network, that are widely accepted (Yeo et al., 2011). These networks are composed of anatomically separated, but functionally connected regions. Recently, a triple-network model including DMN, salience network, and executive control network has been proposed for further investigation (Wang et al., 2022). Numerous studies have recognized the importance of the DMN network in the pathogenesis of disorders of consciousness. This network is composed mostly of associative cortex in the midline including posterior cingulate cortex (praecuneus), medial prefrontal cortex, and medial, lateral and inferior parietal cortex (Broyd et al., 2009). In healthy subjects, the DMN shows increased activity at rest, in the absence of cognitive tasks (Greicius et al., 2003). Furthermore, the DMN is involved in self-referential processing (internal awareness) (Fingelkurts et al., 2016), while the associative cortex on the convexity (mostly frontoparietal network) is associated with external awareness (Laureys et al., 2004; Vanhaudenhuyse et al., 2011). Hypofunctional DMN is seen as a marker of impaired consciousness (Crone et al., 2011; Fernández-Espejo et al., 2012). Impaired internal DMN connectivity is accompanied by reduced connectivity with all other cortical regions and the mediodorsal thalamus, respectively (He et al., 2015).

However, there is evidence of globally impaired functional connectivity within multiple RSNs (Demertzi et al., 2014). Pathologic intrinsic connectivity is characterized by hypoconnectivity or hyperconnectivity patterns in different RSNs. Hypoconnectivity is observed mainly in DMN and frontoparietal associative networks (Cauda et al., 2009). These findings are supported by PET studies showing that preserving a certain level of brain metabolism in the fronto-parietal network can greatly enhance the likelihood of recovering behavioral indicators of consciousness in individuals diagnosed with UWS (Stender et al., 2014).

Moreover, intrinsic functional connectivity strength in many brain regions significantly correlates with consciousness level and recovery outcome (Qin et al., 2015). Among them, DMN and auditory networks have the highest precision in differentiating DOC patients from healthy controls (85.3%) and VS from MCS patients (>80%) (Demertzi et al., 2014, 2015). On the other hand, increased connectivity (hyperconnectivity) is observed in the brainstem areas, cerebellum, and some limbic structures (Di Perri et al., 2013; Chen et al., 2018). Not only that intrinsic connectivity (in specific RSNs) is disturbed, but there is also abnormal interaction between different RSNs. Different degrees of consciousness impairment are associated with specific connectivity patterns between RSNs clinical DOC symptoms of DOC changes between MCS and VS, followed by further weakness of the functional connectivity, and resulting in two connections systems becoming inhibitory altogether in VS (Di Perri et al., 2013; Chen et al., 2018). Dysfunctional connectivity in VS is characterized by changes in network correlations, appearance of pathological network correlations, and pathological imbalance between positive and negative correlations in network (Di Perri et al., 2018).

Another approach to global network analysis is connectome-based. Brain networks have a topological organization, consisting of nodes that are grouped into modules, while extensively connected nodes are called the “hubs” of the connectome. The hubs play a critical part in coordinating communication between distant parts of the brain (Rawls et al., 2022). The higher complexity of networks is associated with higher levels of consciousness (Varley et al., 2020). However, DOCs are characterized not only by lower system complexity but there is evidence of whole-brain topological reorganization (Coppola et al., 2022). The level of consciousness deterioration is associated to decrease of integration in sensory and cognitive related RSNs, segregation decreases and increases in centrality for sensory-related RSNs (Martínez et al., 2020).

Considering both local (intrinsic) and global network alterations, recent studies proposed that regions sensorimotor integration of high arrangement play a crucial part in supporting consciousness. The view is supported by evidence of a significantly reduced number of connections in the sensorimotor cortex and their correlation with levels of consciousness (Qin et al., 2021). Furthermore, stimulus-related fMRI studies using the auditory, visual, or somatosensory paradigm demonstrated activation in the primary sensory areas (lower-level), but without activation of higher-level associative zones that process external signals (Kotchoubey et al., 2013). This corroborates the hypothesis that loss of consciousness might correlate with the disruption of higher-order associative cortices (Amico et al., 2017).

### EEG findings in DOC

Frequency band analysis provides valuable insights into assessing consciousness. Studies have revealed decreased alpha power and increased delta power in patients with the UWS compared to those in the MCS (Fingelkurts et al., 2012). Furthermore, research has linked specific frequency bands to brain network measures and their association with consciousness. Initial investigations focused on the alpha and beta bands, which are implicated in conscious interactions, self-referential thoughts, internal attention, and sensory-motor processing (Fingelkurts et al., 2012). Connectivity alterations within the DMN occur in the alpha and beta bands among patients with DOC (Fingelkurts et al., 2012). UWS patients exhibit reduced connectivity compared to MCS patients in these frequency bands, regardless of the etiology of brain damage (Fingelkurts et al., 2013). Notably, strong connectivity within alpha frontoparietal networks is correlated with the level of consciousness. In the ß1 band, UWS patients show decreased functional connectivity, particularly in the interhemispheric frontoparietal network (Cacciola et al., 2019). Additionally, UWS patients demonstrate smaller functional connectivity in the alpha and gamma bands, while gamma-band connectivity strength correlates with behavioral responsiveness (Naro et al., 2018). Conversely, MCS patients exhibit connectivity in both short- and long-range networks across different frequency bands (Cacciola et al., 2019). Theta and delta bands are less useful for differential diagnosis in DOC; however, higher cortical functional connectivity in the delta-theta band has been observed in MCS compared to UWS, serving as a robust indicator of conscious states (Rizkallah et al., 2019). In summary, frequency bands play a crucial role in assessing consciousness, with distinct connectivity patterns observed between UWS and MCS patients in different frequency bands (Supplementary material).

The absence of consciousness in patients in the VS is paralleled by impairment in the overall EEG operational architecture (Lehembre et al., 2012; Fingelkurts et al., 2013; Bourdillon et al., 2020). Neuronal assemblies in these patients become smaller, their lifespan is shortened, and they become highly unstable and functionally disconnected (desynchronized). On the other hand, patients in the MCS show a partial restoration of EEG operational architecture, with increased size, lifespan, and stability of neuronal assemblies, as well as an increased number and strength of functional connections among them (Fingelkurts et al., 2013).

Brain networks have traditionally been analysed in anatomical space, but recent research has highlighted the importance of considering the topological aspects of brain networks. Further studies explored even more detailed frameworks to evaluate functional connectivity, for example using multiplex and multilayer network analyses of frequency-specific and area-specific networks (Naro et al., 2021). Alterations in brain networks are not limited to global changes but also manifest in specific subnetworks or regions. It is found that the level of consciousness is associated with the DMN subnetwork. Patients with UWS and MCS display decreased connectivity within the DMN, which is partly attributed to impaired structural connectivity and compromised white matter integrity (Fingelkurts et al., 2012). In addition to the DMN, the frontoparietal (FP) networks play a crucial role in behavioral responsiveness, as measured by the CRS-R, in patients with DOC (Cacciola et al., 2019). Selective disruptions in FP regions are observed in UWS patients compared to MCS patients, indicating a breakdown of long-range connections in favor of shorter connections. This disruption impairs multisensory integration and top-down control processes (Wu et al., 2022). The properties of EEG network topology can differentiate between patients with UWS and MCS at a group level (Cacciola et al., 2019). However, the correlation between network topology measures and behavioral responsiveness, as measured by the CRS-R, is generally weak (Cacciola et al., 2019). Furthermore, functional network switching in DOC occurs at multiple time scales. Cai et al. demonstrated that network switching in the alpha band shows a significant correlation with consciousness levels, particularly for transitions of community assignments (Cai et al., 2020). The DOC brain exhibits a dynamic balance between segregation and integration (Cai et al., 2020). Regarding sensorimotor areas, passive hand movements induce slight desynchronization over the contralateral motor cortex in patients with DOC, suggesting functional reactivity despite network disruption and isolation of the motor areas in UWS patients (Formaggio et al., 2020). Moreover, Zhang et al. conducted a microstate-based study and found that networks in DOC patients exhibit impaired global information processing (network integration) and increased local information processing (network segregation) compared to controls (Zhang et al., 2023). Decreased integration, which reflects functional connectivity between distant areas, is associated with lower levels of consciousness (Zhang et al., 2023).

### Diagnostic role of neuromodulation protocols

Neuromodulation protocols could be used for diagnostic or therapeutic purposes. In this review, we will primarily discuss their diagnostic value. Several methods have been used to access functional connectivity after stimulation protocols. We can divide them into two categories: (a) non-invasive – which is transcranial magnetic stimulation (TMS), and (b) invasive: spinal cord stimulation (SCS) (Bai et al., 2017), vagus nerve stimulation (VNS), and DBS (Arnts et al., 2022; Dang et al., 2023).

The invasive techniques are used primarily for therapeutic reasons. Since it is the most common, non-invasive, and painless technique, we will discuss TMS in more detail. The main advantage of TMS-evoked EEG responses compared to resting-state FC is “active probing” of effective connectivity. In contrast to resting state EEG measurements, TMS-EEG measures and maps cortical excitability and reactivity. Three types of TMS can be distinguished: single-pulse activated once every few seconds, paired-pulse, where two pulses are activated out of phase to inhibit or excite neurons of one hemisphere or to inhibit in one while exciting in the other hemisphere, and repetitive TMS (rTMS), where pulses are sent in fast sequence (Galletta et al., 2011). It has been showed previously that high-frequency rTMS increases the excitability of cortical neurons, while low-frequency rTMS decreases their excitability. Mentioned effects continues throughout the stimulation period (Liu et al., 2018; Guo et al., 2019; Tian and Izumi, 2022). By researching the articles, we found 16 studies with TMS stimulation, including all types of stimulation protocols with different stimulation targets: left primary motor area (M1), supplementary motor area, prefrontal cortex, cerebellum, etc. (Naro et al., 2015, 2016c). Finally, research groups used different types of measurements after modulation protocols: involving clinical assessment (CSR-R) and EEG absolute power spectra and functional measurements (Bai et al., 2018; Han et al., 2022) or post-stimulus time histogram (Naro et al., 2016a), and even neuroimaging (fMRI and PET) in some studies (Lin et al., 2019).

The first observation a significantly different effect of TMS treatment comparing VS and MCS patients; there was an improvement in EEG functional connectivity and increases in power spectra in the majority of MCS patients but modest or no effect in VS patients (Carrière et al., 2020; Hermann et al., 2020; Peng et al., 2022). Therefore, the patients are also called responders and non-responders (Hermann et al., 2020; Peng et al., 2022). Second, some may probably have significant detachment between behavioral and neuroimaging because of serious motor deterioration, rather than a functional cortico-cortical connectivity malfunction (Naro et al., 2016b; Hermann et al., 2020). And third, improvement in EEG functional connectivity parameters correlated well with CRS-R clinical examination scores (Naro et al., 2015).

DBS has been shown to have significant effects on functional connectivity in patients with MCS (Arnts et al., 2022). A study by Arnts et al. found that DBS is associated with changes in functional connectivity and neural variability in MCS patients. The study demonstrated that DBS with a lower frequency and larger volume of activation was associated with a stronger increase in functional connectivity and neural variability (Arnts et al., 2022). This increase in functional connectivity was observed across all frequency bands and throughout the brain, suggesting a widespread reorganization of brain networks [1]. Additionally, Dang et al. showed that DBS improved EEG functional connectivity in patients with MCS, leading to enhanced brain networks and improved consciousness activities (Dang et al., 2023). These findings highlight the positive impact of DBS on functional connectivity in MCS patients. However, enhanced functional connectivity does not necessarily imply overall behavioral improvement (Dang et al., 2023).

However, these results must be interpreted cautiously because of the small patient samples (Zhang et al., 2020). Among other neuromodulation techniques, VNS acts in a bottom-up manner, as opposed to top-down manner techniques (like TMS) in DOC patients (Corazzol et al., 2017; Vitello et al., 2023).

## Discussion

Functional connectivity studies in DOC have predominantly relied on neuroimaging techniques, with a particular focus on resting-state fMRI (Cauda et al., 2009; Crone et al., 2011; Vanhaudenhuyse et al., 2011; Fernández-Espejo et al., 2012; Demertzi et al., 2014; He et al., 2015). Resting-state fMRI has the advantage of being mature and widely available, making it a convenient choice for investigating functional connectivity in DOC patients (Demertzi et al., 2014; Qin et al., 2015). However, it is important to note that fMRI captures hemodynamic signals and cannot directly measure fast neural oscillations, which limits its ability to determine certain aspects of neural activity. Rhythmic neuronal interactions can be quantified using multiple metrics, each with their own advantages and disadvantages. The choice of which metric to use is challenging, as the literature provides numerous options with varying levels of accessibility and comparability between studies. This makes it challenging for researchers to select and justify the most appropriate metric for their study. Furthermore, the algorithmic implementation of a particular interaction metric can vary across research groups, leading to limited accessibility and comparability between studies. These factors contribute to the potential for over-interpretation of results. Additionally, determining causality in detecting true interactions is crucial, as it significantly impacts the interpretation of brain function (Fingelkurts et al., 2012; Kotchoubey et al., 2013; Amico et al., 2017; Qin et al., 2021). Despite these challenges, functional connectivity studies using fMRI have provided valuable insights into the neural mechanisms of DoC.

Initial fMRI studies revealed disruptions in functional connectivity in regions such as the basal ganglia, thalamus, and frontal cortex, shedding light on the structural basis of functional disconnection underlying these conditions. Specifically, using RSNs, studies have shown local hypoconnectivity within multiple RSNs, particularly in the DMN and frontoparietal associative networks, suggesting a breakdown in the coordination and communication between different brain regions critical for normal cognitive functioning (Cauda et al., 2009; Crone et al., 2011; Vanhaudenhuyse et al., 2011; Fernández-Espejo et al., 2012; Demertzi et al., 2014; He et al., 2015; Qin et al., 2015).

In addition to hypoconnectivity, DOC patients also exhibit local hyperconnectivity in certain regions, including limbic structures, brainstem areas, and the cerebellum. This abnormal increase in connectivity may reflect compensatory mechanisms or maladaptive processes in the brain. Abnormal interactions between different RSNs have also been observed, and these abnormal connectivity patterns have been correlated with levels of consciousness, providing valuable information about the severity of the condition and potential prognosis. Furthermore, fMRI studies have demonstrated whole-brain topological reorganization in DoC patients, particularly affecting sensory-related RSNs. This reorganization is characterized by diminished global information processing (network integration) and increased local information processing (network segregation).

In addition to fMRI, electrophysiological techniques (particularly EEG) have also been used to estimate functional connectivity in DoC (Fingelkurts et al., 2012, 2013; Naro et al., 2018; Cacciola et al., 2019; Rizkallah et al., 2019; Bourdillon et al., 2020). Although MEG has some advantages over EEG, we focus primarily on EEG studies because of limited research in DOCs. EEG offers high temporal resolution and is cost-effective, making it a promising tool in this field. Frequency band analysis shows altered power and connectivity in specific bands, such as decreased alpha power and increased delta power in patients with UWS compared to those in the MCS. Connectivity disruptions within the DMN occur in the alpha and beta bands among DOC patients, with strong connectivity within alpha frontoparietal networks correlating with the level of consciousness. It has been observed that patients in UWS exhibit impaired EEG operational architecture, with smaller and desynchronized neuronal assemblies, while MCS patients show a partial restoration of EEG operational architecture, with larger and more stable neuronal assemblies and increased functional connections (Lehembre et al., 2012; Bourdillon et al., 2020; Cai et al., 2020; Formaggio et al., 2020; Naro et al., 2021; Wu et al., 2022; Zhang et al., 2023). These alterations extend to specific brain networks, including the DMN and FP networks, and have an impact on network topology and functional connectivity. Impaired global and local information processing is observed in DOC patients, with decreased network integration and increased network segregation compared to controls.

These findings emphasize the importance of frequency-specific connectivity patterns, EEG organizational changes, and network dynamics in assessing consciousness and differentiating between DoC states. Future research aims to determine levels of functional connectivity as biomarkers for responsiveness to potential neuromodulatory interventions.

## Conclusion

Connectomics and network neuroscience provide valuable quantitative frameworks for analysing dynamic brain connectivity, offering new insights into the pathophysiology of disorders of consciousness. By integrating neuroimaging and electrophysiological techniques, functional connectivity studies hold promise for enhancing diagnostic accuracy, guiding treatment approaches, and assessing prognosis in DOC patients. Functional connectivity studies, particularly those conducted using resting-state paradigms, have significantly contributed to our understanding of the underlying neural mechanisms in DOC. These studies have revealed disruptions in functional connectivity, abnormal interactions between networks, and whole-brain topological reorganization.

However, several challenges need to be addressed, such as accurately extracting real signals from artifacts and irrelevant data arising from complex mathematical algorithms. To mitigate these challenges, it is essential to maintain a grounded understanding of basic neuroanatomy and neurophysiology to avoid misinterpretation of the data.

Considering that a significant number of patients exhibit a higher degree of preserved consciousness than clinically classified, functional connectivity techniques can aid clinicians in avoiding misdiagnosis. Expanding research on EEG functional connectivity, given its low-cost and routine implementation, has the potential to become a new gold standard for evaluating cortical integrity, particularly due to its strong correlation with clinical CRS-R testing. However, it should be noted that improved functional connectivity observed after various neuromodulatory interventions does not necessarily imply the full restoration of consciousness levels.

In DOC both awakenees and awareness are significantly impaired. Thalamocortical as well as cortico-cortical dysconnectivity play important role in the pathogenesis of DOC. Furthermore, there is critical role of high-order sensorimotor integration in supporting consciousness. The evidence of preserved sensorimotor integration in the higher-order cortex may hint at the potential for brain recovery. Additional research is required to validate these assumptions.

## Author contributions

GP designed the study and wrote the first version of the manuscript. GP, MR, VD, and DC conducted the literature research, contributed to the data analysis, study concept, and design. VD and DC interpreted the results and revised the manuscript. All authors read and approved the final version of the manuscript as submitted.

## Funding

This research was supported by the Croatian Science Foundation project CSF-IP-2020-02-4308, DC.

## Conflict of interest

The authors declare that the research was conducted in the absence of any commercial or financial relationships that could be construed as a potential conflict of interest.

## Publisher’s note

All claims expressed in this article are solely those of the authors and do not necessarily represent those of their affiliated organizations, or those of the publisher, the editors and the reviewers. Any product that may be evaluated in this article, or claim that may be made by its manufacturer, is not guaranteed or endorsed by the publisher.
