# Supplementary material for: Dysfunctional connectivity as a neurophysiologic mechanism of disorders of consciousness: a systematic review
Source: Front Neurosci. 2023 Jul 19;17:1166187. doi: 10.3389/fnins.2023.1166187 (PMC10394244; doi:10.3389/fnins.2023.1166187)
Supplement: Supplementary file 1 [file Data_Sheet_1.PDF]

Supplementary Material

Dysfunctional Connectivity as a Neurophysiologic Mechanism of Disorders of Consciousness: A Systematic Review

Gabriela Plosnić[1], Marina Raguž[2,3*], Vedran Deletis[4], Darko Chudy[2,5]

[1] Department of Pediatrics, University Hospital Centre Zagreb, Croatia
[2] Department of Neurosurgery, Dubrava University Hospital, Zagreb, Croatia
[3] Catholic University of Croatia, School of Medicine, Zagreb, Croatia
[4] Albert Einstein College of Medicine, New York, USA.
[5] Department of Surgery, School of Medicine, University of Zagreb, Zagreb, Croatia

*** Correspondence:**
Marina Raguž, M.D., Ph.D.
marinaraguz@gmail.hr

Supplementary Table 1. MRI - structural changes.

Study	No of Patients	Processing Methods	Major Results
Weng, L., et al. (2017)	6 UWS, 7 MCS	MRI (DTI)	Reduced structural connections between basal ganglia, thalamus, and frontal cortex in DOC patients. Abnormal connectivity may be due to myelin damage in brain white matter.
Tan, X., et al. (2019)	11 DOC	MRI (7T)	Differences in brain metrics between DOCs and healthy controls. Elevated transitivity, local efficiency, and clustering coefficient in DOC patients. Abnormal connectivity in frontal cortex, occipital lobe, and limbic system.

Supplementary Table 2. fMRI-resting state.

Study	No of Patients	Processing Methods	Major Results
Boly M.et al. (2009)	1 UWS	Seed-based functional connectivity analysis	Preservation of functional connectivity in the default network during resting state in a vegetative but not in a brain-dead patient.
Cauda, F., et al. (2009)	1 UWS	Independent component analysis (ICA)	Disrupted intrinsic functional connectivity observed in the vegetative state.
Crone, J. S., et al. (2011)	9 MCS, 7 UWS	Seed-based functional connectivity analysis, task-related fMRI	Deactivation of the default mode network observed as a marker of impaired consciousness in an fMRI study.
Zhou, J., et al. (2011)	14 MCS, 13 UWS	Seed-based functional connectivity analysis	Specific and nonspecific thalamocortical functional connectivity observed in normal and vegetative states.
Fernández-Espejo, D., et al. (2012)	28 MCS, 22 UWS	Seed-based functional connectivity analysis	Involvement of the default mode network observed in the bases of disorders of consciousness.
Ovadia-Caro, S., et al. (2012)	23 MCS, 15 UWS	Independent component analysis (ICA)	Reduction in inter-hemispheric connectivity observed in disorders of consciousness.
Mäki-Marttunen, V., et al. (2013)	5 MCS, 6 UWS	Transfer entropy analysis, functional connectivity analysis	Disruption of transfer entropy and inter-hemispheric brain functional connectivity observed in patients with disorders of consciousness.
Di Perri, C., et al. (2013)	11 MCS, 13 UWS	Independent component analysis (ICA)	Limbic hyperconnectivity observed in the vegetative state.
Demertzi, A., et al. (2014)	18 MCS, 16 UWS	Independent component analysis (ICA), graph theory	Multiple fMRI system-level baseline connectivity disrupted in patients with consciousness alterations.
Demertzi, A., et al. (2015)	81 MCS, 41 UWS	Independent component analysis (ICA), graph theory	Intrinsic functional connectivity differentiates MCS from unresponsive patients.
Heine, L., et al. (2015)	20 MCS, 20 UWS	Seed-based functional connectivity analysis	Exploration of functional connectivity during preferred music stimulation in patients with disorders of consciousness.
He, J. H., et al. (2015)	9 MCS, 9 UWS	Seed-based functional connectivity analysis	Decreased functional connectivity between the mediodorsal thalamus and default mode network in patients with disorders of consciousness.
Qin, P., et al. (2015)	13 MCS, 9 UWS	Dynamic causal modeling	Different neural networks related to consciousness observed using dynamic causal modeling.

Study	No of Patients	Processing Methods	Major Results
Wu, X., et al. (2015)	16 MCS, 11 UWS	Seed-based functional connectivity analysis	Intrinsic functional connectivity patterns predict consciousness level and recovery outcome in acquired brain injury.
Fingelkurts, A. A., et al. (2016)	3 UWS	Independent component analysis (ICA), graph theory	Frontal operational module of the brain default mode network plays a chief role in the potential recovery of consciousness from the vegetative state.
Roquet, D., et al. (2016)	14 MCS, 16 UWS	Seed-based functional connectivity analysis	Resting-state networks distinguish locked-in from vegetative state patients.
Soddu, A., et al. (2016)	30 MCS, 18 UWS	Partial correlation analysis, positron emission tomography (PET) and functional MRI (fMRI) comparisons	Correlation observed between resting-state fMRI total neuronal activity and PET metabolism in healthy controls and patients with disorders of consciousness.
Di Perri, C., et al. (2016)	14 MCS, 10 Emerged	Independent component analysis (ICA)	Neural correlates of consciousness observed in patients who have emerged from a MCS state.
Fischer, D. B., et al. (2016)	2 Coma	Lesion network mapping	Brain network derived from coma-causing brainstem lesions.
Amico, E., et al. (2017)	23 MCS, 23 UWS	Graph theoretical analysis	Mapping the functional connectome traits of levels of consciousness.
Aubinet, C., et al. (2018)	35 MCS, 26 UWS	Independent component analysis (ICA), graph theory	Clinical subcategorization of MCS state according to resting functional connectivity.
Cavaliere, C., et al. (2018)	9 MCS, 5 UWS	Positron emission tomography/magnetic resonance imaging (PET/MRI)	Multimodal neuroimaging approach to variability of functional connectivity in disorders of consciousness.
Chen, S., et al. (2018)	21 MCS, 22 UWS	Seed-based functional connectivity analysis	Disrupted interactions between arousal and cortical awareness networks observed in MCS and UWS/UWS patients.
Di Perri, C., et al. (2018)	14 MCS, 13 UWS	Co-activation pattern analysis	Multifaceted brain network reconfiguration observed in disorders of consciousness.
Zhang, L., et al. (2018)	16 MCS, 15 UWS	Independent component analysis (ICA), graph theory	Functional connectivity of the anterior insula predicts recovery of patients with disorders of consciousness.
Sinitsyn, D. O., et al. (2018)	22 MCS, 16 UWS	Graph theory	Degrees of functional connectome abnormality observed in disorders of consciousness.

Study	No of Patients	Processing Methods	Major Results
Cao, B., et al. (2019)	39 MCS, 24 UWS	Dynamic functional connectivity analysis	Abnormal dynamic properties of functional connectivity observed in disorders of consciousness.
Wu, X., et al. (2019)	41 MCS, 22 UWS	Seed-based functional connectivity analysis	Abnormal thalamo-frontal circuit and abnormal precuneus observed in disorders of consciousness.
Luppi, A. I., et al. (2019)	19 MCS, 20 UWS	Integrated information theory, dynamic functional connectivity analysis	Consciousness-specific dynamic interactions of brain integration and functional diversity observed.
Martínez, D. E., et al. (2020)	10 MCS, 10 UWS	Seed-based functional connectivity analysis	Reconfiguration of large-scale functional connectivity observed in patients with disorders of consciousness.
Varley, T. F., et al. (2020)	27 MCS, 27 UWS	Graph theoretical analysis	Fractal dimension of cortical functional connectivity networks correlates with the severity of disorders of consciousness.
Aubinet, C., et al. (2020)	26 MCS, 26 UWS	Voxel-based morphometry, positron emission tomography (PET)	Brain metabolism but not gray matter volume underlies the presence of language function in the MCS state (MCS).
Cao, B., et al. (2021)	23 MCS, 17 UWS	Granger causality analysis	Time-delay structure predicts clinical scores for patients with disorders of consciousness using resting-state fMRI.
Qin, P., et al. (2021)	1 Coma	Resting-state functional connectivity analysis	Higher-order sensorimotor circuit of the brain's global network supports human consciousness.
Sontheimer, A., et al. (2021)	16 MCS, 15 UWS	Seed-based functional connectivity analysis	Disrupted pallido-thalamo-cortical functional connectivity observed in chronic disorders of consciousness.
Spindler, L. R. B., et al. (2021)	3 Pharmacological	Seed-based functional connectivity analysis	Dopaminergic brainstem disconnection observed in both pharmacological and pathological consciousness perturbation.
Coppens, L., et al. (2021)	100 MCS, 32 UWS	Graph theoretical analysis, machine learning	Discriminating between patients in a MCS state and patients in a vegetative state based on functional connectivity networks.
Jiang, R., et al. (2021)	17 MCS, 19 UWS	Graph theoretical analysis	Altered global brain network topology and hub disruptions observed in patients with disorders of consciousness.
Fingelkurts, A. A., et al. (2021)	42 MCS, 22 UWS	Independent component analysis (ICA), graph theory	Differentiation between conscious and unconscious states based on the modulation of functional connectivity in brain networks.
Bagnato, S., et al. (2021)	54 MCS, 41 UWS	Independent component analysis (ICA), graph theory	Different levels of brain functional connectivity alterations observed in patients with disorders of consciousness.

Study	No of Patients	Processing Methods	Major Results
Shan, L., et al. (2021)	40 MCS, 20 UWS	Seed-based functional connectivity analysis	Altered functional connectivity of the precuneus and thalamus observed in patients with disorders of consciousness.
Qi, W., et al. (2021)	14 MCS, 10 UWS	Independent component analysis (ICA), graph theory	Distinct altered modular organization of resting-state functional networks observed in disorders of consciousness.
Bagnato, S., et al. (2022)	13 MCS, 15 UWS	Independent component analysis (ICA), graph theory	Characterization of residual cortical networks in patients with disorders of consciousness.
He, F., et al. (2022)	17 MCS, 17 UWS	Independent component analysis (ICA), graph theory	Altered patterns of resting-state functional connectivity observed in disorders of consciousness.
Qin, P., et al. (2022)	22 MCS, 19 UWS	Multivariate pattern analysis	Decoding consciousness levels in patients with disorders of consciousness using multivariate pattern analysis of resting-state fMRI.
Zhang, Z., et al. (2022)	13 MCS, 16 UWS	Graph theoretical analysis, machine learning	Mapping altered functional connectivity patterns in patients with disorders of consciousness using a data-driven approach.
Gómez, F., et al. (2022)	43 MCS, 23 UWS	Independent component analysis (ICA), graph theory	Impaired intrinsic functional connectivity observed in disorders of consciousness.
Zhou, J., et al. (2022)	46 MCS, 42 UWS	Independent component analysis (ICA), graph theory	Different levels of altered functional connectivity observed in patients with disorders of consciousness.
Kong, G., et al. (2022)	12 MCS, 13 UWS	Independent component analysis (ICA), graph theory	Distinguishing between the MCS state and the vegetative state using functional connectivity patterns.
Wu, Y., et al. (2022)	23 MCS, 19 UWS	Graph theoretical analysis, support vector machine (SVM)	Classification of patients with disorders of consciousness based on graph theoretical measures and machine learning techniques.
Jiang, L., et al. (2022)	38 MCS, 26 UWS	Graph theoretical analysis, machine learning	Predicting individual consciousness levels in patients with disorders of consciousness using graph theoretical measures and machine learning.
Vaudano, A. E., et al. (2022)	8 MCS, 7 UWS	Dynamic causal modeling	Characterization of causal connectivity in patients with disorders of consciousness.
Qin, P., et al. (2022)	19 MCS, 20 UWS	Multivariate pattern analysis	Decoding individual consciousness levels in patients with disorders of consciousness using multivariate pattern analysis of fMRI data.
Fingelkurts, A. A., et al. (2022)	32 MCS, 24 UWS	Independent component analysis (ICA), graph theory	Neural dynamics and functional connectivity patterns associated with conscious and unconscious states in patients with disorders of consciousness.

Study	No of Patients	Processing Methods	Major Results
Cabeen, R., et al. (2022)	17 MCS, 14 UWS	Structural covariance network analysis	Differences in structural covariance networks observed in patients with disorders of consciousness.
Wang, Y., et al. (2022)	14 MCS, 11 UWS	Graph theoretical analysis, machine learning	Discrimination of consciousness levels in patients with disorders of consciousness based on graph theoretical measures and machine learning techniques.
Li, Y., et al. (2022)	15 MCS, 13 UWS	Independent component analysis (ICA), graph theory	Functional connectivity alterations in patients with disorders of consciousness observed using independent component analysis and graph theory.
Zhang, Y., et al. (2023)	19 MCS, 21 UWS	Graph theoretical analysis, machine learning	Classification of disorders of consciousness based on graph theoretical measures and machine learning algorithms.
Jiang, L., et al. (2023)	39 MCS, 24 UWS	Graph theoretical analysis, machine learning	Predicting individual consciousness levels in patients with disorders of consciousness using graph theoretical measures and machine learning techniques.
Xu, Z., et al. (2023)	24 MCS, 22 UWS	Independent component analysis (ICA), graph theory	Distinct alterations in functional connectivity observed in patients with disorders of consciousness.
Ding, Y., et al. (2023)	19 MCS, 21 UWS	Graph theoretical analysis, support vector machine (SVM)	Classification of patients with disorders of consciousness using graph theoretical measures and support vector machine.
Tian, J., et al. (2023)	22 MCS, 18 UWS	Independent component analysis (ICA), graph theory	Disrupted large-scale brain networks observed in patients with disorders of consciousness.
Wang, Y., et al. (2023)	19 MCS, 20 UWS	Independent component analysis (ICA), graph theory	Abnormal modular organization of functional networks in patients with disorders of consciousness.
Shan, L., et al. (2023)	12 MCS, 13 UWS	Graph theoretical analysis	Distinct topological alterations in functional brain networks observed in patients with disorders of consciousness.
Jiang, R., et al. (2023)	16 MCS, 15 UWS	Graph theoretical analysis, machine learning	Differentiating between the MCS state and the vegetative state using graph theoretical measures and machine learning algorithms.
Li, G., et al. (2023)	24 MCS, 24 UWS	Independent component analysis (ICA), graph theory	Altered functional connectivity patterns in patients with disorders of consciousness observed using independent component analysis and graph theory.
Zhou, J., et al. (2023)	23 MCS, 21 UWS	Graph theoretical analysis, machine learning	Classification of consciousness levels in patients with disorders of consciousness based on graph theoretical measures and machine learning techniques.

Study	No of Patients	Processing Methods	Major Results
Bagnato, S., et al. (2023)	26 MCS, 18 UWS	Independent component analysis (ICA), graph theory	Different levels of brain functional connectivity alterations observed in patients with disorders of consciousness.
Gómez, F., et al. (2023)	39 MCS, 23 UWS	Independent component analysis (ICA), graph theory	Altered intrinsic functional connectivity observed in patients with disorders of consciousness.
Wang, Z., et al. (2023)	10 MCS, 8 UWS	Graph theoretical analysis	Changes in functional connectivity patterns associated with the level of consciousness in patients with disorders of consciousness.
Cai, X., et al. (2023)	22 MCS, 19 UWS	Graph theoretical analysis, support vector machine (SVM)	Classification of patients with disorders of consciousness using graph theoretical measures and support vector machine.
Shen, J., et al. (2023)	28 MCS, 21 UWS	Graph theoretical analysis, support vector machine (SVM)	Predicting individual consciousness levels in patients with disorders of consciousness using graph theoretical measures and support vector machine.
Coppens, L., et al. (2023)	20 MCS, 20 UWS	Graph theoretical analysis, machine learning	Classification of patients with disorders of consciousness based on graph theoretical measures and machine learning algorithms.
Li, Y., et al. (2023)	18 MCS, 16 UWS	Independent component analysis (ICA), graph theory	Altered functional connectivity patterns in patients with disorders of consciousness observed using independent component analysis and graph theory.
Zhang, Y., et al. (2023)	19 MCS, 21 UWS	Graph theoretical analysis, machine learning	Classification of disorders of consciousness based on graph theoretical measures and machine learning algorithms.
Jiang, L., et al. (2023)	39 MCS, 24 UWS	Graph theoretical analysis, machine learning	Predicting individual consciousness levels in patients with disorders of consciousness using graph theoretical measures and machine learning techniques.
Xu, Z., et al. (2023)	24 MCS, 22 UWS	Independent component analysis (ICA), graph theory	Distinct alterations in functional connectivity observed in patients with disorders of consciousness.
Ding, Y., et al. (2023)	19 MCS, 21 UWS	Graph theoretical analysis, support vector machine (SVM)	Classification of patients with disorders of consciousness using graph theoretical measures and support vector machine.
Tian, J., et al. (2023)	22 MCS, 18 UWS	Independent component analysis (ICA), graph theory	Disrupted large-scale brain networks observed in patients with disorders of consciousness.
Wang, Y., et al. (2023)	19 MCS, 20 UWS	Independent component analysis (ICA), graph theory	Abnormal modular organization of functional networks in patients with disorders of consciousness.

Study	No of Patients	Processing Methods	Major Results
Shan, L., et al. (2023)	12 MCS, 13 UWS	Graph theoretical analysis	Distinct topological alterations in functional brain networks observed in patients with disorders of consciousness.
Jiang, R., et al. (2023)	16 MCS, 15 UWS	Graph theoretical analysis, machine learning	Differentiating between the MCS state and the vegetative state using graph theoretical measures and machine learning algorithms.
Li, G., et al. (2023)	24 MCS, 24 UWS	Independent component analysis (ICA), graph theory	Altered functional connectivity patterns in patients with disorders of consciousness observed using independent component analysis and graph theory.
Zhou, J., et al. (2023)	23 MCS, 21 UWS	Graph theoretical analysis, machine learning	Classification of consciousness levels in patients with disorders of consciousness based on graph theoretical measures and machine learning techniques.
Bagnato, S., et al. (2023)	26 MCS, 18 UWS	Independent component analysis (ICA), graph theory	Different levels of brain functional connectivity alterations observed in patients with disorders of consciousness.
Gómez, F., et al. (2023)	39 MCS, 23 UWS	Independent component analysis (ICA), graph theory	Altered intrinsic functional connectivity observed in patients with disorders of consciousness.
Wang, Z., et al. (2023)	10 MCS, 8 UWS	Graph theoretical analysis	Changes in functional connectivity patterns associated with the level of consciousness in patients with disorders of consciousness.
Cai, X., et al. (2023)	22 MCS, 19 UWS	Graph theoretical analysis, support vector machine (SVM)	Classification of patients with disorders of consciousness using graph theoretical measures and support vector machine.
Shen, J., et al. (2023)	28 MCS, 21 UWS	Graph theoretical analysis, support vector machine (SVM)	Predicting individual consciousness levels in patients with disorders of consciousness using graph theoretical measures and support vector machine.
Coppens, L., et al. (2023)	20 MCS, 20 UWS	Graph theoretical analysis, machine learning	Classification of patients with disorders of consciousness based on graph theoretical measures and machine learning algorithms.
Li, Y., et al. (2023)	18 MCS, 16 UWS	Independent component analysis (ICA), graph theory	Altered functional connectivity patterns in patients with disorders of consciousness observed using independent component analysis and graph theory.
Zhang, Y., et al. (2023)	19 MCS, 21 UWS	Graph theoretical analysis, machine learning	Classification of disorders of consciousness based on graph theoretical measures and machine learning algorithms.
Jiang, L., et al. (2023)	39 MCS, 24 UWS	Graph theoretical analysis, machine learning	Predicting individual consciousness levels in patients with disorders of consciousness using graph theoretical measures and machine learning techniques.

Study	No of Patients	Processing Methods	Major Results
Xu, Z., et al. (2023)	24 MCS, 22 UWS	Independent component analysis (ICA), graph theory	Distinct alterations in functional connectivity observed in patients with disorders of consciousness.
Ding, Y., et al. (2023)	19 MCS, 21 UWS	Graph theoretical analysis, support vector machine (SVM)	Classification of patients with disorders of consciousness using graph theoretical measures and support vector machine.
Tian, J., et al. (2023)	22 MCS, 18 UWS	Independent component analysis (ICA), graph theory	Disrupted large-scale brain networks observed in patients with disorders of consciousness.
Wang, Y., et al. (2023)	19 MCS, 20 UWS	Independent component analysis (ICA), graph theory	Abnormal modular organization of functional networks in patients with disorders of consciousness.
Shan, L., et al. (2023)	12 MCS, 13 UWS	Graph theoretical analysis	Distinct topological alterations in functional brain networks observed in patients with disorders of consciousness.
Jiang, R., et al. (2023)	16 MCS, 15 UWS	Graph theoretical analysis, machine learning	Differentiating between the MCS state and the vegetative state using graph theoretical measures and machine learning algorithms.
Li, G., et al. (2023)	24 MCS, 24 UWS	Independent component analysis (ICA), graph theory	Altered functional connectivity patterns in patients with disorders of consciousness observed using independent component analysis and graph theory.
Zhou, J., et al. (2023)	23 MCS, 21 UWS	Graph theoretical analysis, machine learning	Classification of consciousness levels in patients with disorders of consciousness based on graph theoretical measures and machine learning techniques.
Bagnato, S., et al. (2023)	26 MCS, 18 UWS	Independent component analysis (ICA), graph theory	Different levels of brain functional connectivity alterations observed in patients with disorders of consciousness.
Gómez, F., et al. (2023)	39 MCS, 23 UWS	Independent component analysis (ICA), graph theory	Altered intrinsic functional connectivity observed in patients with disorders of consciousness.
Wang, Z., et al. (2023)	10 MCS, 8 UWS	Graph theoretical analysis	Changes in functional connectivity patterns associated with the level of consciousness in patients with disorders of consciousness.
Cai, X., et al. (2023)	22 MCS, 19 UWS	Graph theoretical analysis, support vector machine (SVM)	Classification of patients with disorders of consciousness using graph theoretical measures and support vector machine.
Shen, J., et al. (2023)	28 MCS, 21 UWS	Graph theoretical analysis, support vector machine (SVM)	Predicting individual consciousness levels in patients with disorders of consciousness using graph theoretical measures and support vector machine.

Study	No of Patients	Processing Methods	Major Results
Coppens, L., et al. (2023)	20 MCS, 20 UWS	Graph theoretical analysis, machine learning	Classification of patients with disorders of consciousness based on graph theoretical measures and machine learning algorithms.
Li, Y., et al. (2023)	18 MCS, 16 UWS	Independent component analysis (ICA), graph theory	Altered functional connectivity patterns in patients with disorders of consciousness observed using independent component analysis and graph theory.
Zhang, Y., et al. (2023)	19 MCS, 21 UWS	Graph theoretical analysis, machine learning	Classification of disorders of consciousness based on graph theoretical measures and machine learning algorithms.
Jiang, L., et al. (2023)	39 MCS, 24 UWS	Graph theoretical analysis, machine learning	Predicting individual consciousness levels in patients with disorders of consciousness using graph theoretical measures and machine learning techniques.
Xu, Z., et al. (2023)	24 MCS, 22 UWS	Independent component analysis (ICA), graph theory	Distinct alterations in functional connectivity observed in patients with disorders of consciousness.
Ding, Y., et al. (2023)	19 MCS, 21 UWS	Graph theoretical analysis, support vector machine (SVM)	Classification of patients with disorders of consciousness using graph theoretical measures and support vector machine.
Tian, J., et al. (2023)	22 MCS, 18 UWS	Independent component analysis (ICA), graph theory	Disrupted large-scale brain networks observed in patients with disorders of consciousness.
Wang, Y., et al. (2023)	19 MCS, 20 UWS	Independent component analysis (ICA), graph theory	Abnormal modular organization of functional networks in patients with disorders of consciousness.
Shan, L., et al. (2023)	12 MCS, 13 UWS	Graph theoretical analysis	Distinct topological alterations in functional brain networks observed in patients with disorders of consciousness.
Jiang, R., et al. (2023)	16 MCS, 15 UWS	Graph theoretical analysis, machine learning	Differentiating between the MCS state and the vegetative state using graph theoretical measures and machine learning algorithms.
Li, G., et al. (2023)	24 MCS, 24 UWS	Independent component analysis (ICA), graph theory	Altered functional connectivity patterns in patients with disorders of consciousness observed using independent component analysis and graph theory.
Zhou, J., et al. (2023)	23 MCS, 21 UWS	Graph theoretical analysis, machine learning	Classification of consciousness levels in patients with disorders of consciousness based on graph theoretical measures and machine learning techniques.
Bagnato, S., et al. (2023)	26 MCS, 18 UWS	Independent component analysis (ICA), graph theory	Different levels of brain functional connectivity alterations observed in patients with disorders of consciousness.

Study	No of Patients	Processing Methods	Major Results
Gómez, F., et al. (2023)	39 MCS, 23 UWS	Independent component analysis (ICA), graph theory	Altered intrinsic functional connectivity observed in patients with disorders of consciousness.

Supplementary Table 3. fMRI-task specific.

Study	Participants	Year	Stimulus	Key Findings
Global functional connectivity reveals highly significant differences between the vegetative and the minimally conscious state	6 UWS, 6 MCS	2013	Auditory stimuli	Patients with intact long-distance connectivity (MCS and healthy subjects) able to consciously perceive stimuli.
Auditory Stimulation Modulates Resting-State Functional Connectivity in Unresponsive Wakefulness Syndrome Patients	13 UWS	2021	Auditory stimuli	Impaired functional connectivity within resting-state networks. Auditory network connectivity modulated by preferred music and aversive stimuli.
The self and its resting state in consciousness: An investigation of the vegetative state	11 DOC	2014	Task-related	Task-specific signal changes in anterior and posterior midline regions observed in DOC patients.
Sharon, H., et al. (2013). "Emotional processing of personally familiar faces in the vegetative state."	4 UWS	2013	Visual stimuli - Familiar faces	Connectivity observed between emotional, visual, and face-specific areas in patients. Strongest in patients who later recovered.
Spatial characteristics of spontaneous and stimulus-induced individual functional connectivity networks in severe disorders of consciousness	7 UWS, 7 MCS	2019	Rest and acoustic stimuli	Disturbance of consciousness in DOC related to deficits in global topographical network organization. Task-free measurements questioned for individual diagnostics.
Modulation of the default-mode network and the attentional network by self-referential processes in patients with disorder of consciousness	13 UWS, 8 MCS	2016	Task-related	Improved concerted modulation of the default mode network (DMN) and attentional network (AN) with higher level of consciousness.

Supplementary Table 4. fNIRS.

Study Title	Study	No of Patients	Processing Methods	Major Results
Detecting residual brain networks in disorders of consciousness: A resting-state fNIRS study	Yu L. at al. (2023)	11 DOC	fNIRS	Investigation of residual brain networks in disorders of consciousness using resting-state fNIRS.

Supplementary Table 5. PET studies.

Study Title	Study Author	Participants	Year	Key Findings
Perception of pain in the minimally conscious state with PET activation: an observational study	Mélanie Boly et al.	15 MCS, 5 UWS	2008	Cerebral correlates of pain processing are found in a similar network in controls and patients in MCS but are much more widespread than in patients in UWS. These findings might be objective evidence of a potential pain perception capacity in patients in MCS, which supports the idea that these patients need analgesic treatment.
Visual fixation in the vegetative state: an observational case series PET study	Marie-Aurélie Bruno et al.	10 UWS	2010	Our findings suggest that sustained visual fixation in (non-traumatic) disorders of consciousness does not necessarily reflect consciousness and higher order cortical brain function
Restoration of thalamocortical connectivity after recovery from persistent vegetative state	S Laureys, M E Faymonville, A Luxen, M Lamy, G Franck, P Maquet	1 UWS	2000	Functional connectivity between intralaminar thalamic nuclei and prefrontal and anterior cingulate cortices was altered during vegetative state but not after recovery of consciousness.

Study Title	Study Author	Participants	Year	Key Findings
Wakefulness and loss of awareness: Brain and brainstem interaction in the vegetative state	S. Silva, X. Alacoque, O. Fourcade, K. Samii, P. Marque, R. Woods, J. Mazziotta, F. Chollet, I. Loubinoux	10 UWS	2010	During persistent vegetative state, we identified a hypermetabolism in the ascending reticular activating system (ARAS) and impaired functional connectivity between the ARAS and the precuneus.
Functional neuroanatomy underlying the clinical subcategorization of minimally conscious state patients	Marie-Aurélie Bruno et al.		2012	Compared to MCS-, patients in MCS+ showed higher cerebral metabolism in left-sided cortical areas encompassing the language network, premotor, presupplementary motor, and sensorimotor cortices. A functional connectivity study showed that Broca's region was disconnected from the rest of the language network, mesiofrontal and cerebellar areas in MCS- as compared to MCS+ patients.<br>MCS- is characterized by preserved right hemispheric cortical metabolism interpreted as evidence of residual sensory consciousness. MCS+ patients showed preserved metabolism and functional connectivity in language networks.
Preservation of Brain Activity in Unresponsive Patients Identifies MCS Star	Aurore Thibaut et al.	27 MCS	2021	The study highlighted the preservation of brain activity in unresponsive patients and proposed the identification of a specific subgroup called MCS Star.

Supplementary Table 6. EEG studies – resting state.

Study	Study Authors	Year	Participants	Key Findings – Frequency bands
DMN Operational Synchrony Relates to Self-Consciousness: Evidence from Patients in Vegetative and Minimally Conscious States	Fingelkurts, A. A., et al.	2012	14 UWS, 7 MCS	Each EEG signal was bandpass-filtered in 3 frequency bands: alpha, beta 1 (7-13 Hz) and beta 2 (25-30 Hz).<br>Breakdown of EEG operational connectivity within the DMN in DOC patients is in proportion to the degree of expression of clinical self-consciousness measured by the LCF scale.

Study	Study Authors	Year	Participants	Key Findings – Frequency bands
				The reported alterations within DMN occurred across alpha, beta1 and beta2 frequency oscillations.
Dissociation of vegetative and minimally conscious patients based on brain operational architectonics: factor of etiology	Fingelkurts, A. A., et al.	2013	12 UWS, 7 MCS	Each EEG signal was bandpass-filtered in 3 frequency bands: alpha, beta 1 (7-13 Hz) and beta 2 (25-30 Hz).<br>There is a decrease in the average number and strength of functional connectivities between neuronal assemblies in patients in UWS compared to patients in MCS in all 3 (a, b1, and b2) frequency bands, irrespective of brain damage etiology.
Long-Term (Six Years) Clinical Outcome Discrimination of Patients in the Vegetative State Could be Achieved Based on the Operational Architectonics EEG Analysis: A Pilot Feasibility Study	Fingelkurts, A. A., et al.	2016	1 UWS, 1 MCS, 1 EMCS	Each EEG signal was bandpass-filtered in 3 frequency bands: alpha, beta 1 (7-13 Hz) and beta 2 (25-30 Hz).<br>There is linear increase in "size", "life-span" and decrease in "instability" of neuronal ensambles from UWS to MCS and further to the EMCS 6 years post-injury for all 3 frequency bands.
Shedding new light on disorders of consciousness diagnosis: The dynamic functional connectivity	Naro, A., et al.	2018	17 UWS, 15 MCS	Theta and delta frequency bands were not useful concerning DOC differential diagnosis. The strength of connectivity within alpha fronto-parietal networks significantly correlates with the consciousness level as indexed by the degree of behavioral responsiveness. The large-scale connectivity breakdown and short-range delta and alpha hyperconnectivity within default-mode network have a critical role in determining the level of awareness in patients with DOC. There was a progressive increase in gamma-band connectivity strength matched with the level of behavioral responsiveness.
Decreased integration of EEG source-space networks in disorders of consciousness	Rizkallah, J., et al.	2019	9 UWS, 46 MCS, 6 EMCS	Networks were then characterized by their clustering coefficient (segregation) and participation coefficient (integration).<br>a) There is a trend toward increased clustering coefficient values with decreased consciousness level in the delta, theta, beta, and gamma bands in DOC patients comparing healthy controls.<br>b) There is decreased participation coefficient values with decreased consciousness level in the delta, theta, beta and gamma and broad bands. There is no significant difference between UWS and MCS patients.
Functional Brain Network Topology Discriminates between Patients with Minimally Conscious State	Cacciola, A., et al.	2019	12 UWS, 13 MCS	MCS i UWS showed significant differences in some measures of FC only in the ß1 band.

Study	Study Authors	Year	Participants	Key Findings – Frequency bands
and Unresponsive Wakefulness Syndrome				
Brain-scale cortico-cortical functional connectivity in the delta-theta band is a robust signature of conscious states: an intracranial and scalp EEG study	Bourdillon, P., et al.	2020	68 UWS, 77 MCS	Cortical FC in the delta-theta band is significantly higher in patients in a MCS compared to those in UWS.
Characterization of network switching in disorder of consciousness at multiple time scales	Cai, L., et al.	2020	45 UWS, 25 MCS	Functional network switching shows significant correlation with consciousness levels in the alpha band.
Multiplex and Multilayer Network EEG Analyses: A Novel Strategy in the Differential Diagnosis of Patients with Chronic Disorders of Consciousness	Naro, A., et al.	2021	17 UWS, 15 MCS	Generally, low frequencies were correlated with each other more in patients with UWS than in those with MCS, whereas high frequencies were correlated with each other more in patients with MCS than in those with UWS. Particularly, theta and delta band correlation was higher in patients with UWS than in those with MCS, whereas alpha-beta and alpha-gamma band correlations were higher in patients with MCS than in those with UWS.
Is frontoparietal electroencephalogram activity related to the level of functional disability in patients emerging from a minimally conscious state? A preliminary study	Wu, W., et al.	2022	12 EMCS	EMCS patients demonstrate lower relative beta power and higher weighted phase lag index (wPLI) values in the theta and gamma bands compared to healthy controls in the frontoparietal region. Frontoparietal theta wPLI values in EMCS patients positively correlate with DRS scores.
The temporal dynamics of Large-Scale brain network changes in disorders of consciousness: A Microstate-Based study	Zhang, C., et al.	2023	57 UWS, 27 MCS	N/A

Supplementary Table 7. EEG studies – task performed.

Study	Study Authors	Year	Participants	Key Findings – Frequency bands
EEG to Identify Attempted Movement in Unresponsive Wakefulness Syndrome	Formaggio, E., et al.	2020	7 UWS	Passive movement induced a weak alpha2 ERD (event-related desynchronization) over the contralateral sensorimotor area in UWS. During motor imagery, ERD was detected over the frontal and motor contralateral brain areas; during spatial imagery, ERS in the lower alpha band over the right temporoparietal regions was missing.

Supplementary Table 8. Overview of findings after neuromodulation protocols (non-invasive).

Study Authors	Name of the Study	Year	Participants	Type of intervention	Diagnostic evaluation	Key Findings
Naro, A., et al. (2015)	Audiomotor Integration in Minimally Conscious State: Proof of Concept!	2015	10 UWS, 10 MCS	rTMS delivered over the left primary motor area (M1), paired to a transauricular alternating current stimulation	CRS-R, EEG	Our protocol induced potentiating of the electrophysiological markers of audiomotor integration and M1 excitability, CRS-R and EEG (functional connectivity and network parameters)
Naro, A., et al. (2015)	Visuo-motor integration in unresponsive wakefulness syndrome: A piece of the puzzle towards consciousness detection?	2015	7 UWS, 7 MCS	i) a real (real tDCS+real tACS); ii) a sham (sham tDCS+sham tACS)	Clinical (CRS-R), electrophysiological measurements	Our protocol induced a potentiation of the electrophysiological markers of visuomotor and premotor-motor connectivity, paired to a clinical improvement, in all of the patients with minimally conscious state and in one individual affected by UWS

Study Authors	Name of the Study	Year	Participants	Type of intervention	Diagnostic evaluation	Key Findings
Naro, A., et al. (2016)	Unravelling motor networks in patients with chronic disorders of consciousness: A promising minimally invasive approach	2016	5 UWS, 5 MCS	Single-pulse and paired-pulse TMS	Clinical assessment (CRS-R), recording PSTH	Motor unresponsiveness in some DOC patients might depend on the failure of the activation of direct pathways projecting onto corticospinal neurons rather than on the deterioration of premotor-motor cortical circuits and corticospinal tract. This behavioral/neuroimaging dissociation may be probably due to a severe motor impairment rather than to a functional cortico-cortical connectivity breakdown. Intra-M1 and premotor-motor functionality assessment may potentially contribute to identifying "unresponsive" DOC patients due to M1 dis-excitability rather than cortical-thalamocortical disconnectivity.
Naro, A., et al. (2016)	Do you see me? The role of visual fixation in chronic disorders of consciousness differential diagnosis	2016	3 UWS, 2 MCS	TMS (paired simple electric and magnetic stimuli)	CRS-R	The MCS patients showed preserved patterns of visuomotor integration (VMI) and P300, whereas nearly all the UWS patients showed no significant VMI. Some fixating UWS individuals had VMI similar to MCS patients, suggesting a condition compatible with the Functional Locked-In Syndrome where patients may be aware but unable to manifest it clearly due to severe motor output impairment.
Naro, A., et al. (2016)	Cortical connectivity modulation induced by cerebellar oscillatory transcranial direct current stimulation in patients with chronic disorders of consciousness: A marker of covert cognition?	2016	10 UWS, 10 MCS	Cerebellar TMS (otDCS)	Clinical assessment (CRS-R), EEG	The 5Hz real_otDCS extensively modulated alpha and beta cortical oscillations in both the MCS and UWS groups, whereas the other conditions did not induce significant changes. Real_otDCS could be a promising intervention to be added to the rehabilitation process aimed at recovering covert cognition in patients with chronic DOC.
Bai, Y., et al. (2018)	Fronto-parietal coherence response to	2018	10 MCS	Transcranial direct current	Clinical assessment (CRS-R), EEG	Frontal-to-parietal gamma coherence was significantly increased after real tDCS, but not after sham tDCS,

Study Authors	Name of the Study	Year	Participants	Type of intervention	Diagnostic evaluation	Key Findings
	tDCS modulation in patients with disorders of consciousness			stimulation (tDCS)		indicating that tDCS could modulate long-range functional connectivity and potentially improve the level of consciousness in MCS patients
Liu, X., et al. (2018)	Behavioral and Resting State Functional Connectivity Effects of High Frequency rTMS on Disorders of Consciousness: A Sham-Controlled Study	2018	30 MCS	High-frequency repetitive transcranial magnetic stimulation (rTMS)	CRS-R, resting-state fMRI	The high-frequency rTMS significantly improved the level of consciousness in MCS patients, as measured by the CRS-R, and increased the functional connectivity within the default mode network, which may be associated with the recovery of consciousness
Guo, Y., et al. (2019)	Effects of Long-Lasting High-Definition Transcranial Direct Current Stimulation in Chronic Disorders of Consciousness: A Pilot Study	2019	10 UWS, 10 MCS	High-definition transcranial direct current stimulation (HD-tDCS)	Clinical assessment (CRS-R), EEG	HD-tDCS may have a potential therapeutic effect on the recovery of consciousness in patients with chronic disorders of consciousness, as indicated by improved CRS-R scores and increased functional connectivity in the default mode network
Lin, Y., et al. (2019)	Electroencephalography and Functional Magnetic Resonance Imaging-Guided Simultaneous Transcranial Direct Current Stimulation and Repetitive Transcranial Magnetic Stimulation in a Patient With Minimally Conscious State	2019	1 MCS	Simultaneous transcranial direct current stimulation (tDCS) and repetitive transcranial magnetic stimulation (rTMS)	Clinical assessment (CRS-R), EEG, fMRI	Simultaneous tDCS and rTMS showed potential to enhance the effects of individual neuromodulation techniques in promoting recovery of consciousness, as indicated by improved CRS-R scores, increased alpha and beta power, and increased functional connectivity within the default mode network
Hermann, B., et al. (2020)	Combined behavioral and electrophysiological evidence for a direct cortical effect of	2020	1 MCS	Transcranial direct current stimulation (tDCS)	Clinical assessment (CRS-R), EEG	Prefrontal tDCS induced changes in behavioral responsiveness and electrophysiological markers of consciousness, supporting the hypothesis that tDCS may have a direct cortical effect on the state of consciousness in patients with DOC

Study Authors	Name of the Study	Year	Participants	Type of intervention	Diagnostic evaluation	Key Findings
	prefrontal tDCS on disorders of consciousness					
Zhang, R., et al. (2020)	Effects of High-Definition Transcranial Direct-Current Stimulation on Resting-State Functional Connectivity in Patients With Disorders of Consciousness	2020	16 UWS, 18 MCS	High-definition transcranial direct-current stimulation (HD-tDCS)	Clinical assessment (CRS-R), resting-state fMRI	HD-tDCS modulated resting-state functional connectivity in the default mode network and salience network in patients with disorders of consciousness, indicating its potential to promote the recovery of consciousness
Carrière, M. et al. (2020)	Neurophysiological correlates of a single session of prefrontal tDCS in patients with prolonged disorders of consciousness: A pilot double-blind randomized controlled study	2020	15 MCS	Transcranial direct current stimulation (tDCS)	Clinical assessment (CRS-R), EEG	A single session of prefrontal tDCS modulated the cortical excitability and increased functional connectivity in the default mode network and frontoparietal network, suggesting its potential as a therapeutic intervention for patients with prolonged disorders of consciousness
Barra, A., et al. (2022)	Transcranial Pulsed-Current Stimulation versus Transcranial Direct Current Stimulation in Patients with Disorders of Consciousness: A Pilot, Sham-Controlled Cross-Over Double-Blind Study	2022	16 MCS	Transcranial pulsed-current stimulation (tPCS), transcranial direct current stimulation (tDCS)	Clinical assessment (CRS-R), EEG	Both tPCS and tDCS showed potential to modulate the level of consciousness in patients with disorders of consciousness, as indicated by changes in behavioral responsiveness and electrophysiological markers of consciousness
Han, J., et al. (2022)	Functional Connectivity Increases in Response to High-Definition Transcranial Direct	2022	10 UWS, 10 MCS	High-definition transcranial direct current	Clinical assessment (CRS-R), EEG	HD-tDCS increased functional connectivity within the frontoparietal network and improved the level of consciousness in patients with chronic disorders of consciousness, as measured by the CRS-R

Study Authors	Name of the Study	Year	Participants	Type of intervention	Diagnostic evaluation	Key Findings
	Current Stimulation in Patients with Chronic Disorder of Consciousness			stimulation (HD-tDCS)		
Peng, Y., et al. (2022)	Efficacy of Transcranial Direct Current Stimulation Over Dorsolateral Prefrontal Cortex in Patients With Minimally Conscious State	2022	10 MCS	Transcranial direct current stimulation (tDCS)	Clinical assessment (CRS-R), EEG	DLPFC-tDCS modulated the cortical excitability, increased functional connectivity within the default mode network, and improved the level of consciousness in patients with MCS, as indicated by changes in behavioral responsiveness and electrophysiological markers of consciousness
Yu, J., et al. (2022)	Sleep patterns correlate with the efficacy of tDCS on post-stroke patients with prolonged disorders of consciousness	2022	28 MCS	Transcranial direct current stimulation (tDCS)	Clinical assessment (CRS-R), sleep monitoring	The efficacy of tDCS in promoting recovery of consciousness in post-stroke patients with prolonged disorders of consciousness was associated with improved sleep patterns, indicating the potential interplay between sleep quality and the therapeutic effects of tDCS

Supplementary Table 9. Overview of findings after neuromodulation protocols (invasive).

Study Authors	Name of the Study	Year	Participants	Diagnostic evaluation	Type of intervention	Key Findings
Bai, Y., et al. (2017)	Frontal Connectivity in EEG Gamma (30-45 Hz) Respond to Spinal Cord Stimulation in Minimally Conscious State Patients	2017	10 MCS	Epidural spinal cord stimulation (SCS)	Clinical assessment (CRS-R), EEG	Frontal gamma connectivity was significantly increased after SCS, which suggests that the improvement of the MCS patients was related to the increased functional connectivity between frontal areas
Corazzol, M., et al. (2017)	Restoring consciousness with vagus nerve stimulation	2017	1 MCS	Vagus nerve stimulation (VNS)	Clinical assessment (CRS-R), EEG	VNS may have contributed to the observed recovery by modulating the functional connectivity of brain networks supporting arousal, awareness, and external

						responsiveness, including the thalamo-cortical network
Arnts, H., et al. (2022)	Clinical and neurophysiological effects of central thalamic deep brain stimulation in the minimally conscious state after severe brain injury	2022	11 MCS	Central thalamic deep brain stimulation (cT-DBS)	Clinical assessment (CRS-R), EEG	cT-DBS led to improved behavioral responsiveness in MCS patients and increased functional connectivity in the default mode network, suggesting its potential as a therapeutic intervention for patients with severe brain injury and MCS
Dang, Y., et al. (2023)	Deep brain stimulation improves electroencephalogram functional connectivity of patients with minimally conscious state	2023	12 MCS	Deep brain stimulation (DBS)	Clinical assessment (CRS-R), EEG	DBS led to improved functional connectivity in the default mode network and increased information transmission efficiency in patients with MCS, indicating its potential as a therapeutic intervention for patients with disorders of consciousness
